# Draft Genome Sequence of a Fungus (Fusarium tricinctum) Cultured from a Monoisolate Native to the Himalayas

**DOI:** 10.1128/genomeA.00365-18

**Published:** 2018-05-17

**Authors:** Narendra Meena, M. Vasundhara, M. Sudhakara Reddy, Prashanth Suravajhala, U. S. Raghavender, Krishna Mohan Medicherla

**Affiliations:** a Department of Biotechnology and Bioinformatics, Birla Institute of Scientific Research, Jaipur, Rajasthan, India; b Department of Biotechnology, Thapar Institute of Engineering & Technology, Patiala, Punjab, India

## Abstract

Here, we report the draft *de novo* genome sequence assembly of Fusarium tricinctum (strain T6), using IonTorrent sequencing chemistry and an Ion 530 chip ExT kit for sequencing. The genome assembly resulted in 42,732,204 bp from a total 6.62 Gb, with a median read length of 386 bp.

## GENOME ANNOUNCEMENT

Fusarium tricinctum (strain T6) was isolated from the bark samples of Taxus baccata L. subsp. wallichiana (Zucc.) Pilger, also known as Himalayan yew, collected from Bhadrewah (district Doda, Jammu and Kashmir, India). We have assembled the genomic data sequenced using IonTorrent S5XL-00694 as single reads into a draft genome sequence. We report the best assembly of the genome, with an approximate size of 42,732,204 bp, comprised of 617 contigs and 13,132 genes. F. tricinctum has been isolated as an endophytic fungus capable of producing different secondary metabolites (1).

The fungus was grown on potato dextrose broth for 3 weeks, and the genomic DNA was obtained from the mycelium according to the method described in van Kan et al. (2). IonTorrent sequencing chemistry and an Ion 530 chip ExT kit were employed for sequencing, which resulted in 42,732,204 bp from a total 6.62 Gb, with a median read length of 386 bp. Raw single-end reads (19,415,463) were trimmed using Trimmomatic (3). Specifically, reads with bases having a Phred score ≤25 were trimmed. This resulted in 18,213,812 high-quality reads being employed for the assembly. We have used two different software tools for assembling the genome, *viz*., Mimicking Intelligent Reads Assembly (MIRA) (4) and St. Petersburg Genome Assembler (SPAdes) (5). We summarize below the core statistics of both the assemblies.

(i) With MIRA, the assembly resulted in 617 contigs totaling 42,732,204 bp (*N*_50_, 177,443 bp; largest scaffold, 1,057,131 bp; ∼100.0× coverage).
(ii) With SPAdes, the assembly resulted in 1,892 contigs totaling 42,821,728 bp (*N*_50_, 182,652 bp; largest scaffold, 803,475 bp; ∼99.9× coverage).

The size of the assembled genome sequence compares well with those of other published genome sequences of Fusarium species (e.g., F. fujikuroi, 43.83 Mb [6]; F. graminearum, 36.44 Mb [7]; F. oxysporum, 61.35 Mb [7]; F. verticillioides, 41.77 Mb [7, 8]; and F. solani, 51.21 Mb [9]). Gene prediction was carried out using AUGUSTUS (version 3.3) (10), with the gene parameters trained from F. graminearum species. The number of genes predicted in the F. tricinctum genome assemblies (MIRA, 13,132, and SPAdes, 13,109) were in good agreement with the total number of genes reported for other Fusarium species, *viz.,* F. fujikuroi, 14,813; F. graminearum, 13,322; F. oxysporum, 20,925; F. verticillioides, 15,869; and F. solani, 15,705. The genome completeness was assessed using Benchmarking Universal Single-Copy Orthologs (BUSCO) (11), which estimated the genome assembly to be 98.9% (MIRA) and 98.7% (SPAdes) complete, based on the presence of conserved single-copy orthologous gene sets specific to Ascomycota. The DOmain-based General Measure for transcriptome and proteome quality Assessment (DOGMA) (12) was employed for assessing the predicted proteome as a percentage of the eukaryotic core set, which comprises 950 single-domain and 493 multidomain conserved domain arrangements (CDAs). MIRA and SPAdes assemblies were observed to be 95.77% complete in terms of single-domain and multidomain CDAs. The genome sequence described here is of particular interest to further understanding the molecular mechanism behind horizontal gene transfer events and why certain species tend to evolve with enzyme profiles from pathogenic/nonpathogenic relatives.

### Accession number(s).

This whole-genome shotgun project is deposited in GenBank under the accession number PTXX00000000.

## References

[1] Ola ARB, Thomy D, Lai D, Brötz-Oesterhelt H, Proksch P 2013 Inducing secondary metabolite production by the endophytic fungus *Fusarium tricinctum* through coculture with *Bacillus subtilis*. J Nat Prod 76:2094–2099. doi:10.1021/np400589h. 24175613

[2] van Kan JAL, van den Ackerveken GFM, De Wit PJGM 1991 Cloning and characterization of the cDNA of avirulence gene *avr9* of the fungal pathogen *Cladosporium fulvum*, causal agent of tomato leaf mold. Mol Plant Microbe Interact 4:52–59. doi:10.1094/MPMI-4-052. 1799694

[3] Bolger AM, Lohse M, Usadel B 2014 Trimmomatic: a flexible trimmer for Illumina sequence data. Bioinformatics 30:2114–2120. doi:10.1093/bioinformatics/btu170. 24695404 PMC4103590

[4] Chevreux B, Pfisterer T, Drescher B, Driesel AJ, Müller WEG, Wetter T, Suhai S 2004 Using the miraEST assembler for reliable and automated mRNA transcript assembly and SNP detection in sequenced ESTs. Genome Res 14:1147–1159. doi:10.1101/gr.1917404. 15140833 PMC419793

[5] Bankevich A, Nurk S, Antipov D, Gurevich AA, Dvorkin M, Kulikov AS, Lesin VM, Nikolenko SI, Pham S, Prjibelski AD, Pyshkin AV, Sirotkin AV, Vyahhi N, Tesler G, Alekseyev MA, Pevzner PA 2012 SPAdes: a new genome assembly algorithm and its applications to single-cell sequencing. J Comput Biol 19:455–477. doi:10.1089/cmb.2012.0021. 22506599 PMC3342519

[6] Wiemann P, Sieber CMK, von Bargen KW, Studt L, Niehaus E-M, Espino JJ, Huß K, Michielse CB, Albermann S, Wagner D, Bergner SV, Connolly LR, Fischer A, Reuter G, Kleigrewe K, Bald T, Wingfield BD, Ophir R, Freeman S, Hippler M, Smith KM, Brown DW, Proctor RH, Münsterkötter M, Freitag M, Humpf H-U, Güldener U, Tudzynski B 2013 Deciphering the cryptic genome: genome-wide analyses of the rice pathogen *Fusarium fujikuroi* reveal complex regulation of secondary metabolism and novel metabolites. PLoS Pathog 9:e1003475. doi:10.1371/journal.ppat.1003475. 23825955 PMC3694855

[7] Cuomo CA, Guldener U, Xu J-R, Trail F, Turgeon BG, Di Pietro A, Walton JD, Ma L-J, Baker SE, Rep M, Adam G, Antoniw J, Baldwin T, Calvo S, Chang Y-L, DeCaprio D, Gale LR, Gnerre S, Goswami RS, Hammond-Kosack K, Harris LJ, Hilburn K, Kennell JC, Kroken S, Magnuson JK, Mannhaupt G, Mauceli E, Mewes H-W, Mitterbauer R, Muehlbauer G, Munsterkotter M, Nelson D, O’Donnell K, Ouellet T, Qi W, Quesneville H, Roncero MIG, Seong K-Y, Tetko IV, Urban M, Waalwijk C, Ward TJ, Yao J, Birren BW, Kistler HC 2007 The *Fusarium graminearum* genome reveals a link between localized polymorphism and pathogen specialization. Science 317:1400–1402. doi:10.1126/science.1143708. 17823352

[8] Ma L-J, van der Does HC, Borkovich KA, Coleman JJ, Daboussi M-J, Di Pietro A, Dufresne M, Freitag M, Grabherr M, Henrissat B, Houterman PM, Kang S, Shim W-B, Woloshuk C, Xie X, Xu J-R, Antoniw J, Baker SE, Bluhm BH, Breakspear A, Brown DW, Butchko RAE, Chapman S, Coulson R, Coutinho PM, Danchin EGJ, Diener A, Gale LR, Gardiner DM, Goff S, Hammond-Kosack KE, Hilburn K, Hua-Van A, Jonkers W, Kazan K, Kodira CD, Koehrsen M, Kumar L, Lee Y-H, Li L, Manners JM, Miranda-Saavedra D, Mukherjee M, Park G, Park J, Park S-Y, Proctor RH, Regev A, Ruiz-Roldan MC, Sain D, Sakthikumar S, Sykes S, Schwartz DC, Turgeon BG, Wapinski I, Yoder O, Young S, Zeng Q, Zhou S, Galagan J, Cuomo CA, Kistler HC, Rep M 2010 Comparative genomics reveals mobile pathogenicity chromosomes in *Fusarium*. Nature 464:367–373. doi:10.1038/nature08850. 20237561 PMC3048781

[9] Coleman JJ, Rounsley SD, Rodriguez-Carres M, Kuo A, Wasmann CC, Grimwood J, Schmutz J, Taga M, White GJ, Zhou S, Schwartz DC, Freitag M, Ma L, Danchin EGJ, Henrissat B, Coutinho PM, Nelson DR, Straney D, Napoli CA, Barker BM, Gribskov M, Rep M, Kroken S, Molnár I, Rensing C, Kennell JC, Zamora J, Farman ML, Selker EU, Salamov A, Shapiro H, Pangilinan J, Lindquist E, Lamers C, Grigoriev IV, Geiser DM, Covert SF, Temporini E, VanEtten HD 2009 The genome of *Nectria haematococca*: contribution of supernumerary chromosomes to gene expansion. PLoS Genet 5:e1000618. doi:10.1371/journal.pgen.1000618. 19714214 PMC2725324

[10] Keller O, Kollmar M, Stanke M, Waack S 2011 A novel hybrid gene prediction method employing protein multiple sequence alignments. Bioinformatics 27:757–763. doi:10.1093/bioinformatics/btr010. 21216780

[11] Simão FA, Waterhouse RM, Ioannidis P, Kriventseva EV, Zdobnov EM 2015 BUSCO: assessing genome assembly and annotation completeness with single-copy orthologs. Bioinformatics 31:3210–3212. doi:10.1093/bioinformatics/btv351. 26059717

[12] Dohmen E, Kremer LPM, Bornberg-Bauer E, Kemena C 2016 DOGMA: domain-based transcriptome and proteome quality assessment. Bioinformatics 32:2577–2581. doi:10.1093/bioinformatics/btw231. 27153665

